# Muscle quality index comparisons between Hispanics and non-Hispanic Caucasians using dual energy X-ray absorptiometry and handgrip strength

**DOI:** 10.1038/s41430-024-01484-y

**Published:** 2024-08-08

**Authors:** Ayush Mehra, Ronald L. Snarr, Kyung-Shin Park, Jessica L. Krok-Schoen, Stefan A. Czerwinski, Brett S. Nickerson

**Affiliations:** 1 School of Health and Rehabilitation Sciences, The Ohio State University, Columbus, OH USA; 2 Department of Kinesiology, Texas A&M University-Corpus Christi, Corpus Christi, TX USA; 3 College of Nursing and Health Sciences, Texas A&M International University, Laredo, TX USA

**Keywords:** Diseases, Nutrition

## Abstract

**Background & Aims:**

Muscle quality index (MQI) can be computed in various ways. Also, many studies have evaluated MQI in older adults and non-Hispanic populations. The aim of this study was to compare various muscle quality indexes between Hispanics and non-Hispanic Caucasians when stratifying grip strength and appendicular lean mass measurements.

**Methods:**

235 participants (aged 25.5 ± 9.5 for males and 26.4 ± 9.9 for females) completed a dual energy X-ray absorptiometry (DXA) scan to assess appendicular lean mass (ALM). Handgrip strength (HGS) was assessed using a handheld dynamometer. MQI was computed using four different models: 1). MQI_RA_: ALM and HGS of right arm and hand, respectively; 2). MQI_LA_: ALM and HGS of left arm and hand, respectively; 3). MQI_ARMS_: ALM and HGS of both arms and hands, respectively; and 4). MQI_TOTAL_: ALM of upper and lower-limbs and HGS of left and right hand.

**Results:**

Hispanic males and females exhibited lower HGS compared to Caucasians with effect sizes ranging from trivial (*d* = 0.17) to moderate (*d* = 0.80). Females demonstrated higher MQI values compared to males for MQI_ARMS_ (*d* = 0.70), MQI_RA_ (*d* = 0.75), and MQI_LA_ (*d* = 0.57). However, MQI_TOTAL_ yielded a small practical effect (*d* = 0.33) in favor of males (3.2 ± 0.5 kg/kg vs. 3.1 ± 0.5 kg/kg). After factoring by sex and ethnicity, Hispanic males and females, compared to non-Hispanic Caucasians males and females, showed trivial-to-small practical differences (*d* values ranging from 0.03 to 0.39).

**Conclusions:**

These results demonstrate MQI models vary across sex, particularly when utilizing models that account for upper extremity strength and ALM (i.e., MQI_ARMS_, MQI_RA_, and MQI_LA_). Lastly, to establish consistency in future research, the present study recommends using MQI models that account for ALM of upper- and lower-limbs (i.e., MQI_TOTAL_). However, research measuring muscular strength via one upper-limb (e.g., left hand) might consider measuring ALM of the corresponding arm (e.g., left arm) when computing muscle quality (e.g., MQI_LA_).

## Introduction

Muscular strength and skeletal muscle tissue exhibits a progressive decline in aging [1, 2]. Moreover, the manifestation of diminished muscular strength and skeletal muscle tissue is characterized by an asynchronous pattern, with the rate of decline in muscle strength surpassing that of skeletal muscle tissue [3]. For example, after 50 years of age, skeletal muscle tissue experiences an annual decline of around 1 to 2 percent, while muscular strength averages a decline of approximately 1.5 percent [4, 5]. Given these changes, it is important to utilize quantitative methods that can be used to monitor muscular strength and skeletal muscle tissue. One technique for monitoring these components in clinical and research testing environments is muscle quality index (MQI), which is characterized by the ratio of muscular strength relative to skeletal muscle tissue [3].

MQI is used to evaluate how efficiently muscle functions relative to its mass [6]. A high MQI is typically associated with better muscle health whereas a low MQI may indicate issues such as muscle wasting or reduced muscle function. Monitoring MQI is advantageous due to its ability to serve as a proxy for physical performance, representing an array of physiological adaptations in response to training. MQI is also pertinent for younger demographics who are interested in monitoring body composition. For example, muscle quality (MQ) may serve as a strategic approach for young adults who are seeking to enhance functional capacity [6]. This is likely attributed to poor MQ, which has previously been found to impact approximately 24% of young adults with severe obesity, and can lead to an increased risk of functional disability and obesity [7]. Altogether, these findings highlight the importance of measuring MQ across various age spectrums, including young adults.

Comparisons of MQI across ethnicity is another area that demands more attention. Unfortunately, a majority of MQI research thus far has been conducted in non-Hispanic populations. As a result, it is difficult to determine whether disparities exist across Hispanic and non-Hispanic ethnicities. Nonetheless, previous research found lower MQI in Hispanic males than non-Hispanic Caucasians [8]. Despite not reaching statistical significance, Lopes et al. [9] have shown females tend to exhibit higher MQI values than males in metrics that incorporate appendicular lean mass of the arms. Contrarily, MQI differences were smaller when comparing males vs. females, and between races/ethnicities (e.g., Hispanics vs. non-Hispanic Caucasians), when including appendicular lean mass measures of all four extremities [9]. Collectively, these discrepancies highlight the need to further evaluate MQI across Hispanic and non-Hispanic populations, as well as sex differences between males and females. Moreover, a comprehensive examination on the differences that occur when computing MQI via different approaches for handgrip strength (i.e., left hand, right hand, or combined sum) and appendicular lean mass measures (e.g., left and right arm(s) or combined upper and lower extremities) needs further understanding. Therefore, the purpose of this study was to compare various muscle quality indexes between Hispanics and non-Hispanic Caucasians when stratifying grip strength and appendicular lean mass measurements.

## Methods

### Participants

This study consisted of Hispanic (n = 131) and non-Hispanic Caucasian (n = 104) adults. Data from two research laboratories (Tuscaloosa, AL and Laredo, TX) were combined for this study. Sample size was determined via G-Power software, which demonstrated a sample size of 67 participants for each ethnicity would be sufficient for a power of 0.60, a significance level of 0.05, and a moderate effect size of 0.60 [10]. Eligible participants were 1) at least 18 years of age; 2) reported no cardiac, pulmonary, or metabolic diseases; 3) < 350 lb. due to dual energy X-ray absorptiometry (DXA) table restrictions; and 4) Hispanic or non-Hispanic Caucasian descent. Exclusion criteria included persons with non-disease related conditions that may affect body composition, intra- and extra-cellular fluid volumes, or DXA measurements (i.e., those currently or recently pregnant, persons with limb amputations, and individuals with implanted metallic devices). All participants provided written informed consent and completed a medical history questionnaire prior to participation in the study. The testing protocol, recruitment flyers, and consent forms were reviewed and approved by the Institutional Review Board at each university.

### Procedures

All research participants reported to the laboratory for data collection following pre-testing guidelines, which included 1) no high-intensity exercise for 24 hours, 2) fasting ≥8 hours, 3) no alcohol or caffeine for ≥24 hours, 4) no water intake ≥2 hours. The adherence to pre-testing guidelines for each participant was assessed via a questionnaire upon arrival to the laboratory. Once pre-testing guideline adherence was ensured, hydration (i.e., urine specific gravity), anthropometric (i.e., height and body mass), body composition (via DXA), and handgrip strength (HGS) assessments were completed. Prior to all anthropometric and body composition measurements, shoes, jewelry, and metallic objects were removed to minimize measurement error. Hydration was assessed via urine specific gravity using a hand-held refractometer (Atago SUR-NE, Atago Corp Ltd., Tokyo, Japan). Participants exceeding a urine specific gravity of 1.029 were asked to reschedule their testing time for another day [11]. Standing height was measured to the nearest 0.1 cm using a stadiometer (SECA 213, Seca Ltd., Hamburg, Germany), while body mass (BM) was measured to the nearest 0.1 kg using a digital scale (Tanita BWB-800, Tanita Corporation, Tokyo, Japan).

### Dual-energy X-ray absorptiometry

Body composition was assessed using DXA (GE Lunar Prodigy; Software version 14.10.022; GE Lunar Corporation, Madison, WI, USA). Prior to each use, the DXA was calibrated according to manufacturer guidelines using a standardized calibration block. Participants were positioned supine on the DXA platform with arms resting along the sides of the body and feet secured with Velcro straps around the ankles to reduce movement for the duration of the scan. Data used in this study included fat mass (kg and %), total lean soft tissue (kg), arm lean soft tissue (kg), and leg lean soft tissue (kg). A trained researcher adjusted regions of interest after each DXA scan.

### Handgrip strength

All handgrip tests were completed using a hydraulic hand dynamometer (Jamar, Performance Health Supply Inc., Cedarburg, WI). Prior to each test, the dynamometer was adjusted so the second, third, fourth and fifth digit of the hand (i.e., proximal interphalangeal joint) was bent 90°. To complete each test, participants were instructed to be in a standing position, hold the dynamometer with the elbow flexed at 90°, and squeeze the dynamometer as hard as possible while avoiding the Valsalva maneuver [12]. Handgrip strength was recorded in kg and the dynamometer was reset to zero prior to the next test. This procedure was repeated with the opposite hand and repeated two additional times. The highest value of the three readings for each hand was added together and recorded as the combined sum [13].

### Muscle quality index

MQI_RA_ (kg/kg) was defined as the ratio between highest HGS of the right hand and DXA-derived appendicular lean mass (ALM) of the right arm. MQI_LA_ (kg/kg) was defined as the ratio between highest HGS of the left hand and DXA-derived ALM of the left arm. MQI_ARMS_ (kg/kg) was defined as the ratio between combined sum of HGS (left hand + right hand) and DXA-derived ALM in both arms (left arm + right arm). MQI_TOTAL_ was established as the ratio between combined sum of HGS (left hand + right hand) divided by DXA-derived ALM in the upper- and lower-limbs (left arm + right arm + left leg + right leg).

### Statistical analyses

All data was analyzed using SPSS^©^ v. 28.0 (IBM Corp., Armonk, NY) with data visualizations created using RStudio^©^ (PBC, Boston, MA, USA) and Microsoft Excel™ (Microsoft Corp., Redmond, WA). Tests of normality and histogram analyses were performed on all outcome variables to determine if normal distributions and outliers were present. If outliers were present (i.e., data points greater than ±3 standard deviations), analyses would be calculated with, and without, outliers to determine their relative influence on group means, standard deviations, confidence intervals, between-group comparisons, and regression model analyses.

To compare the effects of sex (i.e., male and female) and ethnicity (i.e., Hispanic and non-Hispanic Caucasian) on each MQI ratio, a multifactorial analysis of variance, along with adjusted Bonferroni post hoc analyses, was conducted. Values from all outcomes are reported as mean (standard deviation) unless otherwise noted. Using group and subgroup standard deviations, sex- and ethnicity-specific cutoff values to determine ‘low’ and ‘extremely low’ MQI values were calculated. ‘Low’ MQI values were defined as one standard deviation below the relative mean for the group or subgroup; whereas ‘extremely low’ MQI values were calculated using two standard deviations below the relative mean [3, 9, 14].

While *p* values have been included in the results, the American Statistical Association does not recommend using *p* value cutoff points (e.g., <0.05) as the basis for determining meaningfulness or importance of an effect [15]. Thus, Cohen’s *d* effect sizes [16] were calculated (where applicable) to determine the magnitude of difference between subgroups and classified using Hopkin’s scale [17]. The scale of magnitude was as follows: trivial effect <0.20, small effect 0.20 – 0.59, moderate effect 0.60 – 1.19, large effect 1.20 –1.99, and very large effect ≥ 2.0.

To assess the influence of individual predictors on each MQI outcome (MQI_LA_, MQI_RA_, MQI_TOTAL_, MQI_ARMS_), hierarchical multiple regression modeling (via the enter method) was used with age, sex, ethnicity, BMI, waist, hip, and body fat percentage (%Fat) as predictor variables. Each independent variable was entered into the exploratory model based on the strength of correlation of each predictor with the outcome variable, with the highest strength of correlation entered first. Unstandardized (B) and standardized (β) values, B confidence intervals, *r* values, adjusted *R*^*2*^, variable inflation factor (VIF), and standard error of the estimate (SEE) were calculated for each MQI model.

## Results

From the sample, there were 131 Hispanic participants (71 females, 60 males) and 104 non-Hispanic Caucasians (51 females, 53 males). The descriptive statistics for all participants are expressed in Table 1 and represented as mean ± standard deviation (unless otherwise noted). Based on descriptives, anthropometrics, predictor variables, and outcome variables, normality tests and histogram analyses determined homogeneity of variance and no observable outliers of the potential 235 participants; thus, all were included in the statistical analyses.

**Table 1:** Descriptive statistics for Hispanic and non-Hispanic Caucasians for the entire group and factored by sex.

		Age (years)	Height (cm)	Weight (kg)	BMI (kg/m^2^)	BF (%)	Waist (cm)	Hip (cm)
Entire Group	M	25.5 ± 9.5	176.3 ± 7.3	83.0 ± 14.6	26.8 ± 4.8	24.7 ± 8.4	88.9 ± 12.6	102.0 ± 8.2
	F	26.4 ± 9.9	162.5 ± 6.2	67.6 ± 14.6	25.7 ± 5.8	35.7 ± 8.3	82.6 ± 15.3	101.7 ± 11.1
	*p*	0.49	<0.001	<0.001	0.13	<0.001	<0.001	0.83
	*d*	0.09	2.05	1.06	0.20	1.31	0.45	0.03
M	H	28.2 ± 11.5	173.4 ± 6.2	85.4 ± 16.7	28.3 ± 5.1	29.3 ± 7.8	93.9 ± 13.9	103.6 ± 9.3
	NH	22.5 ± 5.3	179.6 ± 7.0	80.4 ± 11.4	25.0 ± 3.7	19.5 ± 5.3	83.3 ± 7.9	100.2 ± 6.5
	*p*	0.001	<0.001	0.07	<0.001	<0.001	<0.001	0.03
	*d*	0.62	0.92	0.34	0.74	1.45	0.93	0.41
F	H	29.9 ± 11.2	160.7 ± 5.8	72.0 ± 16.4	27.9 ± 6.3	39.6 ± 7.5	88.4 ± 16.4	105.7 ± 11.7
	NH	21.6 ± 4.4	165.0 ± 6.0	61.5 ± 8.6	22.6 ± 3.3	30.2 ± 6.1	74.5 ± 8.7	96.2 ± 7.1
	*p*	<0.001	<0.001	<0.001	<0.001	<0.001	<0.001	<0.001
	*d*	0.93	0.73	0.77	1.01	1.35	1.01	0.95

*M* males (n = 113), *F* females (n = 122), *H* Hispanic (n = 131), *NH* non-Hispanic Caucasian (n = 104), *d* Cohen’s *d* effect size, *BMI* body mass index, *BF* body fat percentage.

### Overall descriptives

Regarding descriptives and anthropometrics, males were on average taller and heavier, with a lower %Fat, and larger waist and hip circumference than their female counterparts. When factored by ethnicity, Hispanic males were shorter and heavier, with a greater BMI, %Fat and waist and hip circumference than the non-Hispanic Caucasians within this study. These findings were similar when comparing Hispanic females versus their non-Hispanic Caucasian counterparts. Additionally, Hispanic males and females exhibited lower HGS compared to non-Hispanic Caucasians with effect sizes ranging from trivial (*d* = 0.17) to moderate (*d* = 0.80) (Table 2).

**Table 2:** Handgrip, lean mass, and fat mass values for Hispanic and non-Hispanic Caucasians factored by sex.

		Handgrip Strength (kg)		Lean Mass (kg)		Fat Mass (kg)		Lean Mass:Fat Mass (kg:kg)	
		H	NH	H	NH	H	NH	H	NH
**M**	**LA**	41.3 ± 6.1	45.7 ± 8.1	3.7 ± 0.7	4.0 ± 0.8	1.2 ± 0.5	0.8 ± 0.2	3.6 ± 1.5	5.4 ± 1.6
	***d, p***	0.61, 0.002		0.52, 0.007		1.00, <0.001		1.18, <0.001	
	**RA**	42.8 ± 6.5	48.4 ± 7.4	3.7 ± 0.6	4.2 ± 0.8	1.2 ± 0.5	0.8 ± 0.3	3.6 ± 1.5	5.8 ± 2.0
	***d, p***	0.80, <0.001		0.63, 0.001		1.08, <0.001		1.33, <0.001	
	**Arms Combined**	84.1 ± 11.9	94.0 ± 15.1	7.4 ± 1.3	8.2 ± 1.5	–	–	–	–
	***d, p***	0.73, <0.001		0.58, 0.003					
	**Appendicular**	–	–	26.0 ± 4.1	30.1 ± 4.8	–	–	–	–
	***d, p***			0.93, <0.001					
**F**	**LA**	26.3 ± 5.0	27.3 ± 6.1	2.1 ± 0.4	2.1 ± 0.4	1.5 ± 0.6	1.0 ± 0.3	1.6 ± 0.6	2.2 ± 0.7
	***d, p***	0.17, 0.345		0.18, 0.340		0.85, <0.001		0.81, <0.001	
	**RA**	27.7 ± 5.0	29.2 ± 6.0	2.2 ± 0.4	2.1 ± 0.3	1.5 ± 0.6	1.0 ± 0.3	1.6 ± 0.6	2.2 ± 0.7
	***d, p***	0.27, 0.141		0.14, 0.443		0.87, <0.001		0.91, <0.001	
	**Arms Combined**	54.0 ± 9.6	56.4 ± 11.9	4.3 ± 0.8	4.2 ± 0.7	–	–	–	–
	***d, p***	0.23, 0.216		0.16, 0.381					
	**Appendicular**	–	–	17.7 ± 2.9	18.7 ± 2.7	–	–	–	–
	***d, p***			0.36, 0.052					

*M* Males (n = 113), *F* Females (n = 122), *H* Hispanic (M: n = 60; F: n = 71), *NH* non-Hispanic Caucasian (M: n = 53; F: n = 51), *LA* left arm, *RA* right arm, *Appendicular* arms and legs combined, *Lean Mass* fat-free mass minus bone mineral content, *d* Cohen’s *d* effect size, *p* probability value (set a priori at 0.05).

### Muscle quality indexes

**Fig. 1:**
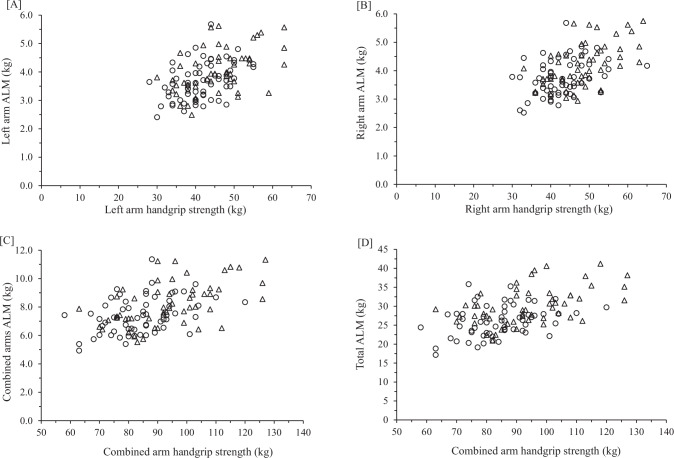
Relationship between handgrip strength (HGS) and appendicular lean mass (ALM) in Hispanic (open circles) and non-Hispanic Caucasian (open triangles) males. **A** Left arm handgrip strength and left arm lean mass; **B** right arm HGS and right arm ALM; **C** combined arms HGS and combined arms ALM; **D** combined arms HGS and total ALM (arms + legs).

**Fig. 2:**
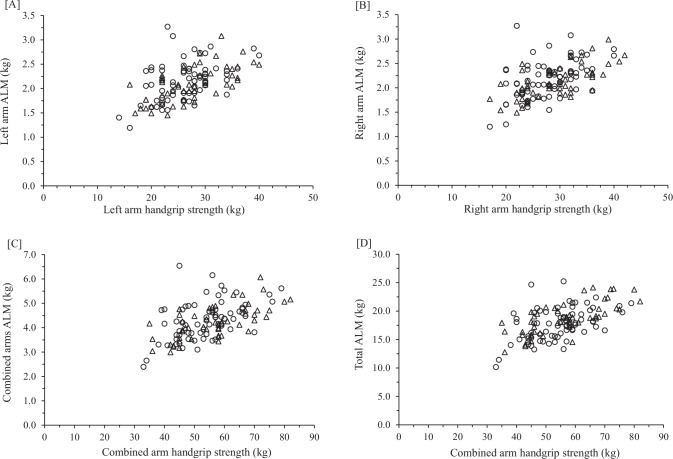
Relationship between handgrip strength (HGS) and appendicular lean mass (ALM) in Hispanic (open circles) and non-Hispanic Caucasian (open triangles) females. **A** Left arm handgrip strength and left arm lean mass; **B** right arm HGS and right arm ALM; **C** combined arms HGS and combined arms ALM; **D** combined arms HGS and total ALM (arms + legs).

When assessing the entire group, females demonstrated higher MQI values compared to males for MQI_ARMS_ (*d* = 0.70), MQI_RA_ (*d* = 0.75), and MQI_LA_ (*d* = 0.57) (Table 3). However, MQI_TOTAL_ yielded a small practical effect (*d* = 0.33) in favor of males (3.2 ± 0.5 kg/kg versus 3.1 ± 0.5 kg/kg). After factoring by sex and ethnicity, Hispanic males, compared to non-Hispanic Caucasian males, showed trivial-to-small practical differences (*d* values ranging from 0.03 to 0.25) for all MQI outcomes (Table 3; Fig. 1). Results were similar when comparing Hispanic females to non-Hispanic Caucasians females, demonstrating trivial-to-small differences (*d* values ranging from 0.15 to 0.39) (Table 3; Fig. 2).

**Table 3:** Muscle Quality Index Values for Hispanic and non-white Hispanic adults.

		MQI_ARMS_				MQI_TOTAL_				MQI_RA_				MQI_LA_			
		Mean (SD)	Low	EL	*d, p*	Mean (SD)	Low	EL	*d, p*	Mean (SD)	Low	EL	*d, p*	Mean (SD)	Low	EL	*d, p*
**All**	**M**	11.6 (1.9)	9.7	7.8	0.70	3.2 (0.5)	2.7	2.2	0.33	11.7 (2.0)	9.7	7.7	0.75	11.5 (2.1)	9.4	7.3	0.57
	**F**	13.0 (2.1)	10.9	8.8	<0.001	3.1 (0.5)	2.6	2.1	0.012	13.3 (2.2)	11.1	8.9	<0.001	12.7 (2.3)	10.4	8.1	<0.001
**M**	**H**	11.6 (1.9)	9.7	7.8	0.04	3.3 (0.5)	2.8	2.3	0.25	11.6 (2.0)	9.6	7.6	0.06	11.5 (2.0)	9.5	7.5	0.03
	**NH**	11.6 (2.0)	9.6	7.6	0.819	3.2 (0.5)	2.7	2.2	0.187	11.8 (1.9)	9.9	8.0	0.734	11.5 (2.2)	9.3	7.1	0.895
**F**	**H**	12.7 (2.1)	10.6	8.5	0.38	3.1 (0.5)	2.6	2.1	0.15	12.9 (2.2)	10.7	8.5	0.39	12.4 (2.2)	10.2	8.0	0.32
	**NH**	13.5 (2.1)	11.4	9.3	0.042	3.0 (0.4)	2.6	2.2	0.402	13.8 (2.1)	11.7	9.6	0.034	13.2 (2.2)	11.0	8.8	0.082

*All* entire group (not factored by ethnicity), *M* Males (n = 113); *F* Females (n = 122), *H* Hispanic (n = 131), *NH* non-Hispanic Caucasian (n = 104), *MQI*_ARMS_ combined handgrip/total arm lean mass, *MQI*_*TOTAL*_ combined handgrip/total appendicular lean mass, *MQI*_*RA*_ right arm handgrip/right arm lean mass, *MQI*_*LA*_ left arm handgrip/left arm lean mass, *SD* standard deviation, *EL* extremely low, MQI’s were defined as “low” if 1 SD below the mean and “extremely low” if 2 SDs below the mean. *d* Cohen’s *d* effect size, *p* probability value (set a priori at 0.05).

For the hierarchical multiple regression models, waist and hip circumferences were removed due to the violation of multicollinearity with BMI (waist variable inflation factor: 12.321; hip variable inflation factor: 6.333). Thus, the final models used BMI, sex, age, ethnicity, and %Fat as predictors for each MQI outcome to reduce multicollinearity. When predicting MQI_ARMS_, the predictor variables demonstrated an R^2^ of 0.27 and SEE of 1.95 kg/kg (*F*(5, 229) = 20.688; *p* = <0.001) indicating low estimation ability of the selected predictor variables. The slope coefficients for age (B = 0.009), %Fat (B = 0.031), BMI (B = −0.233), and ethnicity (B = −0.258) indicated that for each one-unit change in the respective variable (with all others held constant), there was a trivial-to-small increase, or decrease, in predicted MQI_ARMS_. Based on the MQI_ARMS_ model, sex had the highest impact on prediction with a B value of −0.638 indicating that the change in participant entry in the model from female to male reduced estimated MQI by 0.638 kg/kg (Table 4).

**Table 4:** Resultant values of Hierarchical regression predicting muscle quality index in Hispanic (n = 131) and non-Hispanic Caucasian (n = 104) males and females from body mass index, age, sex, ethnicity, and body fat percentage.

				B 95% Confidence Intervals			Correlations		
		Adjusted R^2^	B	Lower	Upper	β	Part	Partial	VIF
MQI_LA_ SEE: ±1.95	Constant	17.503	–	–	–	–	–	–	–
	BMI	0.202	−0.233^a^	−0.310	−0.155	−0.555	−0.366	−0.336	2.733
	Sex	0.256	−0.638	−1.443	0.167	−0.142	−0.103	−0.088	2.584
	Age	0.259	0.009	−0.021	0.039	0.038	0.038	0.033	1.365
	Ethnicity	0.265	−0.258	−0.887	0.370	−0.057	−0.053	−0.046	1.555
	%Fat	0.270	0.031	−0.022	0.084	0.135	0.075	0.064	4.450
MQI_RA_ SEE: ±1.87	Constant	17.585	–	–	–	–	–	–	–
	BMI	0.198	−0.222^a^	−0.296	−0.148	−0.537	−0.364	−0.325	2.733
	Sex	0.292	−0.779^a^	−1.553	−0.006	−0.175	−0.130	−0.109	2.584
	Age	0.294	−0.014	−0.043	0.015	−0.060	−0.062	−0.052	1.365
	Ethnicity	0.298	−0.084	−0.688	0.520	−0.019	−0.018	−0.015	1.555
	%Fat	0.310	0.050	−0.001	0.101	0.224	0.127	0.106	4.450
MQI_ARMS_ SEE: ±1.80	Constant	17.495	–	–	–	–	–	–	–
	BMI	0.212	−0.227^a^	−0.298	−0.156	−0.570	−0.384	−0.345	2.733
	Sex	0.290	−0.706	−1.448	0.037	−0.165	−0.123	−0.103	2.584
	Age	0.287	−0.003	−0.031	0.025	−0.012	−0.012	−0.010	1.365
	Ethnicity	0.291	−0.165	−0.745	0.414	−0.038	−0.037	−0.031	1.555
	%Fat	0.296	0.041	−0.008	0.090	0.192	0.109	0.091	4.450
MQI_TOTAL_ SEE: ±0.44	Constant	4.392	–	–	–	–	–	–	–
	BMI	0.130	−0.048^a^	−0.066	−0.031	−0.519	−0.343	−0.314	2.733
	%Fat	0.133	−0.003	−0.015	0.009	−0.057	−0.031	−0.027	4.450
	Sex	0.173	0.204^a^	0.024	0.384	0.204	0.146	0.127	2.584
	Ethnicity	0.239	−0.301^a^	−0.441	−0.160	−0.298	−0.268	−0.239	1.555
	Age	0.243	0.005	−0.001	0.012	0.102	0.101	0.088	1.365

*MQI*_*LA*_ left arm handgrip/left arm lean mass, *MQI*_*RA*_ right arm handgrip/right arm lean mass, *MQI*_*ARMS*_ combined handgrip/total arm lean mass, *MQI*_*TOTAL*_ combined handgrip/total appendicular lean mass, *SEE* standard error of the estimate, *BMI* body mass index, *%Fat* body fat percentage, *Sex* male vs female, *VIF* variable inflation factor.

^a^Denotes *p* value < 0.05.

Regarding MQI_TOTAL_, regression modeling showed the lowest SEE of any model with 0.44 kg/kg and an adjusted R^2^ of 0.24 (*F*(5, 229) = 16.031; *p* < 0.001). The predictor variable with the largest influence on the model was ethnicity (B = −0.301), indicating that the change from Hispanic to non-Hispanic Caucasian reduced MQI_TOTAL_ by −0.301 kg/kg, with all other variables being held constant. Predictors with the lowest influence included %Fat (B = −0.003), age (B = 0.005), and BMI (B = −0.048).

For MQI_RA_ results exhibited a prediction model with an R^2^ of 0.310 and SEE of 1.87 (*F*(5, 229) = 20.530; *p* < 0.001)). MQI_LA_ showed similar values for the multiple regression model with an R^2^ of 0.27 and SEE of 1.95 (*F*(5, 229) = 16.901; *p* = <0.001). The strongest predictor variable for MQI_RA_ and MQI_LA_ was sex with B values of −0.779 and −0.638, respectively, indicating the change in sex from female to male in the model reduced estimated MQI value by 0.779 kg/kg for MQI_RA_ or 0.638 kg/kg for MQI_LA_.

## Discussion

The purpose of this study was to compare various muscle quality indexes between Hispanics and non-Hispanic Caucasians when stratifying grip strength and appendicular lean mass measurements. Results demonstrated that, on average, Hispanics were shorter, heavier, possessed a greater BMI, %Fat, and waist and hip circumference than non-Hispanic Caucasians. Additionally, Hispanics had a lower HGS than non-Hispanic Caucasians. Despite these differences, there were trivial-to-small differences for all MQI comparisons (i.e., MQI_TOTAL_, MQI_ARMS_, MQI_RA_, and MQI_LA_) between Hispanics and non-Hispanic Caucasians. However, MQI models that only included measurements of the arms (i.e., MQI_ARM_, MQI_RA_, and MQI_LA_) produced larger differences between males and females versus MQI_TOTAL_, which included ALM of the arms and legs. Consequently, this led to sex being a better predictor than ethnicity, based on regression analyses, for MQI_ARMS_, MQI_RA_, and MQI_LA_; whereas ethnicity had the greatest predictability of MQI when incorporating ALM of the arms and legs (i.e., MQI_TOTAL_).

While only a limited number of MQI comparisons between Hispanics and non-Hispanics have been completed, the present study agrees and conflicts with previous research, which is worth further discussion. For instance, when evaluating MQI_TOTAL_, Lopes et al. [9] found Hispanic males and females exhibited slightly higher values (3.5 and 3.4 kg/kg, respectively) than non-Hispanic Caucasians (3.3 and 3.0 kg/kg, respectively). The current study demonstrated nearly identical MQI_TOTAL_ values between Hispanic and non-Hispanic males and females. Additionally, Arajuo et al. [8] found that Hispanic males have lower MQI values than non-Hispanic Caucasians (5.40 and 5.71 kg/kg, respectively) when computing HGS via one hand and the lean mass of both arms. The findings from Arajuo et al. [8] are consistent with the current study, which demonstrated Hispanic females had lower MQI_ARMS_, MQI_RA_, and MQI_LA_ values than non-Hispanic Caucasian. Contrarily MQI_ARMS_, MQI_RA_, and MQI_LA_ values were similar when comparing Hispanic and non-Hispanic Caucasians males in the present study.

The first step in discerning potential differences between current and previous studies includes analyzing the methodological approaches used to compute MQI. For instance, the present study used a GE Lunar Prodigy DXA to estimate body composition, whereas previous work used a Hologic DXA QDR 4500 [8, 9]. Previous research has shown differences between GE and Hologic systems when seeking to estimate body composition [18]. These are important factors to consider as other methodological body composition approaches (e.g., bioimpedance analysis, circumference measures) can be used to estimate ALM and subsequently calculate MQI [19, 20]. In addition to methodological approaches, another explanation for the variance in MQI values exists within the differences of body composition between races and ethnicities. For example, non-Hispanic Caucasian populations tend to have a greater prevalence for sarcopenia and increased fat infiltration of skeletal muscle (i.e., myosteatosis) compared to African Americans [21]. While myosteatosis comparisons of Hispanic adults have yet to be completed, it is plausible that the Hispanic women in the current study possessed higher levels of myosteatosis than non-Hispanic Caucasians, leading to lower MQI values versus their non-Hispanic Caucasian counterparts. Based on these potential rationales for differences in MQI, future studies are encouraged to include a measure of myosteatosis when seeking to compare MQ across various ethnic groups. Additionally, practitioners should be cautious of interpreting MQI results when body composition methods differ across study sites.

The assessment of HGS is also a component that needs to be considered when interpreting MQI findings across studies. For example, the current study used combined HGS (i.e., left hand + right hand) when computing MQI_TOTAL_ and MQI_ARMS_. Moreover, when a single HGS was obtained (e.g., left hand), the corresponding arm (e.g., left arm) was used to compute MQI (e.g., MQI_LA_). This process differs from other studies who have used dominant HGS and both arms for ALM when computing MQI. The reason for using dominant HGS, but both arms for ALM is not entirely clear. One potential rationale may be demonstrated by 10% dominance rules which states that dominant HGS is, on average, 10% greater, then the non-dominant hand [22]. However, research has also shown that non-dominant HGS is equal to, or stronger than, the dominant hand in 28% of subjects [23], with recreational athletes having less profound differences in HGS between hands than non-athletes [24]. Therefore, it is possible the 10% dominance rule once served as a basis when calculating MQI at some point. Nonetheless, the lack of standardization on how to measure MQI likely explains the range of methodological approaches, which makes interpreting results difficult. Until an agreement can be reached when measuring MQI, the current study recommends the following methodological approaches: 1). combined HGS and ALM of both upper extremities (i.e., left arm + right arm); 2) combined HGS and ALM of all 4 extremities (i.e., left arm + right arm + left leg + right left); or 3). single HGS and ALM of corresponding arm. Additionally, it should be noted that the present, and most previous, MQI estimations are calculated using only upper extremity strength (i.e., HGS). By only using upper body strength, generalizations and interpretations are limited, particularly when explaining between sex differences. This is particularly relevant as there are greater differences between sexes when examining muscular strength in the upper body as opposed to the lower body [25]. Therefore, future research should consider MQI models accounting for both upper and lower body strength values as compared to respective ALM.

Notwithstanding the strengths of the present study, it is important to acknowledge the limitations. For example, the current study comprised of young and middle-aged adults. Ideally, the investigation would have comprised of adults across a larger age spectrum to gain a better understand of the relationship of MQI to age. However, it is worth noting that a limited amount of information, regarding MQ, is available in a Hispanic population. Therefore, this study adds to the literature by evaluating MQI in an underrepresented population. Our findings in Hispanic females (i.e., lower MQI_ARMS_, MQI_RA_, and MQI_LA_) also help raise awareness of potential disparities that exist across ethnicities when comparing MQIs. Future studies might investigate this disparity and seek to develop interventions and programs to improve the muscular strength to body composition ratio of Hispanic populations. Another limitation of the current study was not accounting for dominant HGS. Nonetheless, accounting for dominant HGS varies across studies, which is likely attributed to the lack of consensus on estimating MQI. All MQI models of the current study matched HGS with the corresponding arm(s) composition measurements. Thus, future studies might seek to account for dominant hand and determine whether MQI varies across dominant and non-dominant HGS when matched with corresponding arm for ALM.

## Conclusion

The present study sought to complete a comprehensive evaluation of MQI models across sex and race/ethnicity. There are several findings worth nothing: First, the current study found that MQI_TOTAL_ did not differ across sex and ethnicity. Second, muscle quality indexes that only included the arms (MQI_ARMS_, MQI_RA_, and MQI_LA_) yielded larger effect sizes across sex (*d* = 0.57 [MQI_LA_] to 0.75 [MQI_RA_]). Third, the effect size of MQI’s between Hispanic and non-Hispanic Caucasians tended to be slightly larger, albeit small, for females (*d* = 0.15 [MQI_TOTAL_] to 0.39 [MQI_RA_]) than males (*d* = 0.03 [MQI_LA_] to 0.25 [MQI_RA_]). Collectively, these differences demonstrate MQI models vary across sex, particularly when utilizing models that account for upper extremity strength and ALM (i.e., MQI_ARMS_, MQI_RA_, and MQI_LA_). The assessment of muscle quality indexes has varied across previous studies. To be consistent in future research, the present study recommends using MQI models that account for ALM of upper- and lower-extremities (i.e., MQI_TOTAL_). Furthermore, when assessing muscular strength via one upper-extremity (e.g., left hand), researchers and health professionals might consider measuring ALM of the corresponding arm (e.g., left arm) when computing muscle quality (e.g., MQI_LA_).

## Data Availability

Data supporting the findings of this study are available upon a reasonable request. Please contact the corresponding author to access data.
